# Neural Correlates of Successful and Unsuccessful Strategical Mechanisms Involved in Uncertain Decision-Making

**DOI:** 10.1371/journal.pone.0130871

**Published:** 2015-06-18

**Authors:** Julie Giustiniani, Damien Gabriel, Magali Nicolier, Julie Monnin, Emmanuel Haffen

**Affiliations:** 1 Department of Clinical Psychiatry, University Hospital, Besançon, France; 2 EA 481, Laboratory of Neurosciences, University of Franche-Comté, Besançon, France; 3 CIC-1431 Inserm, University Hospital, Besançon, France; Institut Pluridisciplinaire Hubert Curien, FRANCE

## Abstract

The ability to develop successful long-term strategies in uncertain situations relies on complex neural mechanisms. Although lesion studies have shown some of the mechanisms involved, it is still unknown why some healthy subjects are able to make the right decision whereas others are not. The aim of our study was to investigate neurophysiological differences underlying this ability to develop a successful strategy in a group of healthy subjects playing a monetary card game called the Iowa Gambling Task (IGT). In this task, subjects have to win and earn money by choosing between four decks of cards, two were advantageous in the long term and two disadvantageous. Twenty healthy right-handed subjects performed the IGT while their cerebral activity was recorded by electroencephalography. Based on their behavioral performances, two groups of subjects could clearly be distinguished: one who selected the good decks and thus succeeded in developing a Favorable strategy (9 subjects) and one who remained Undecided (11 subjects). No neural difference was found between each group before the selection of a deck, but in both groups a greater negativity was found emerging from the right superior frontal gyrus 600 ms before a disadvantageous selection. During the processing of the feedback, an attenuation of the P200 and P300 waveforms was found for the Undecided group, and a P300 originating from the medial frontal gyrus was found in response to a loss only in the Favorable group. Our results suggest that undecided subjects are hyposensitive to the valence of the cards during gambling, which affects the feedback processing.

## Introduction

Decision-making is a complex cognitive process used in solving open and risky problems in daily life, whose outcome is unpredictable [1,2]. Schematically this decision-making process can be reduced to 3 phases: 1/ Choice evaluation (anticipation); 2/ Selection; 3/ Feedback processing [3]. During choice evaluation, subjects construct scenarios in which long-term consequences and benefits are compared and evaluated to ultimately anticipate what would be the most advantageous choice. Then the consequences of decisions/actions are evaluated by calculating their potential cost and reward during the feedback processing. An impaired decision-making process has harmful direct consequences on social and personal aspects of daily life. Impaired decision-making has been observed in many neuropsychiatric disorders [4–6], such as in behavioral addictions [7] and addictions to substances [8–10]. The first reported observations of impaired decision-making with preserved intellectual abilities were made on subjects with VentroMedial PreFrontal Cortex lesions (VMPFC) [11–13]. To provide a neural explanation for this impairment the somatic marker hypothesis was proposed [1,14]. Based on this hypothesis, external or internal stimuli can produce changes in the body and the brain, changes that contribute to emotions. Briefly, the somatic marker concept refers to the collection of signals related to the body and the brain that characterize the emotional and affective responses. The experiences learned in life are connected to somatic markers that can be used for the prediction of future outcomes in similar situations [15]. The VMPFC is considered as being the cerebral framework that integrates the representations of somatic markers, automatically and implicitly. In cases of uncertain events, the VMPFC guides decision-making toward the most advantageous choices by the generation of the somatic markers [16]. A dysfunction of the VMPFC results in an inability to use somatic markers and therefore leads to impairment in evaluating different options.

The Iowa Gambling Task (IGT) was designed to assess impaired decision-making under conditions of uncertainty by simulating real life economic decisions [11]. The IGT is a monetary card game in which four decks are presented to the subjects. Two of these decks are monetarily advantageous in the long term whereas the other two decks are disadvantageous. This test differs from other neuropsychological tests because it involves emotional processing in addition to a cognitive processing [17,18]. The task involves a long series of gains and losses and to guide their decision towards the decks that they consider as being the most advantageous, the subjects have to follow a long exploratory process [19,20]. This process can be divided in four phases. The first is the pre-punishment phase and corresponds to the period where the subject had still not experience a net loss. From the second to the third phases, pre-hunch and hunch phases, subjects develop an intuition relative to the advantageous or disadvantageous characteristics of each deck. Here, the somatic markers engendered by the emotional component help to anticipate long-term positive and negative outcomes. Most subjects reach the conceptual phase, which corresponds to the period when they have an explicit knowledge of each deck and can elaborate a conscious strategy [17]. Reaching a conceptual phase does not necessarily imply that the strategy will be successful since some patients with VMPFC dysfunction reach this phase but seem to be guided by immediate prospects, which only leads to a global decreased performance in the IGT.

In order to objectivize the relationship between the IGT and somatic markers, this neuropsychological test has been coupled with neurophysiological recordings. The initial reported studies measured the skin conductance responses (SCRs), and showed an increased sympathetic arousal before selecting disadvantageous compared to advantageous decks [8,9] which shows evidence of an anticipatory process during the IGT. In relationship to these studies, neuroimaging has been used to identify the various neural structures involved during decision-making. Many studies using functional Magnetic Resonance Imaging (fMRI) have highlighted the role of VMPFC in information processing [16] and in the generation of the anticipatory component in decision-making [21]. To accurately assess the time course of neural activations of decision-making, electroencephalography (EEG) has been used to complement fMRI, due to its high temporal resolution and because it offers direct access to neuronal signaling [22]. Two information processing correlates have been observed: one around 200–300 ms [3,23–26] and one occurring later around 300–600 ms [3,23,26,27]. During anticipation a slow cortical potential called the Decision Preceding Negativity (DPN) has been observed over the right anterior electrodes 500 ms before selection of a deck [28].

Although several studies have used IGT to describe the neurophysiological differences between a pathological and a healthy population [8–11,26] none has yet described which neuronal mechanisms leads to a favorable or an unfavorable strategy within a same healthy population. However, it would be erroneous to think that the entire healthy population is always able to elaborate the best strategy under uncertain conditions, as proven by our good and bad decisions made in daily life. In fact, several studies have reported up to 37% failure in healthy population in the IGT [8,29–31]. In terms of intellectual performances and socio-demographics data, no differences were found between healthy subjects with Favorable versus poor strategy [8]. However no reported study has been carried out to supply an explanation about the cerebral processes that could explain these differences of performances. In these types of studies, the populations assessed are usually based according to their respective pathologies or their neuropsychological characteristics. The IGT is then used to compare their behavioral or neurophysiological differences. The heterogeneity of the performances within a same population can hardly be taken into account and investigated.

In the present study, our aim was to assess a neurophysiological level that differentiates subjects who are able to develop a long-term favorable strategy from those who are unable to develop a strategy. Therefore, to achieve this goal a population of 20 healthy volunteers performed a version of the IGT adapted to Event-Related Potential (ERP) study, and were divided in different groups based on their performance of this task. To study the neural mechanisms underlying the anticipatory and information processing stages, a recording of the brain activity with EEG during the IGT was performed. The strategic differences on a brain activity level were analyzed for each sub-group. We focused on three main potentials: the DPN, P200 and P300 and assumed that at least one of these potentials would be related to the development of a favorable strategy. Source imaging was used to determine the brain regions involved in the generation of these differences.

## Materials and Methods

### Participants

Twenty healthy right-handed subjects, 10 male and 10 female (mean age = 38.7; SD = 18.3; range 21–59), participated in the study. All participants had no previous medical history of psychiatric disorders, substance abuse, alcohol abuse, neurological diseases, traumatic brain injury or stroke and did not take any medication.

Participants received information regarding the aim and procedures of the experiment, and gave their written informed consent to participate in the study. The influence of real money playing a significant role on motivation, subjects received information that the monetary payment would be proportional to the global gain obtained in the game with an exchange rate of 1% [32–34]. Due to ethic considerations and whatever their performance, all participants received the maximum amount of 75€ at the end of the experiment. The protocol was approved by the Ethics Committee of Besançon University Hospital (authorization given by the General Health Administration (ANSM B90927-60).

In the first part of the study participants had to complete a questionnaire. Its purpose was to better identify the population sample and to ensure the absence of any disorder or personality traits that could affect IGT performance. The psychometric assessment included self-rating scales measuring pathological gambling with the South Oaks Gambling Screen [35–37]; alcohol dependence with the AUDIT [38]; noxious use of cannabis with the DETC/CAGE [38]; addiction to nicotine with the Fagerström test [38]; the existence and the intensity of depressive symptoms with the Beck Depression Inventory abbreviated version [39–41]; anxiety with the Liebowitz scale [42]; impulsivity with the Barratt Impulsiveness Scale (version 10) [43,44]; and individual differences in the five personality dimensions using the Big Five Inventory-French (BFI-Fr) [45–47].

### Experimental task

The task was an electronic version of the IGT [11], adapted for the ERP study and the analysis of brain activity sources. The aim of the task was to win as much money as possible by making successive selections between four decks [11,12].

The composition of decks, values and schedules reward / punishment were predetermined identically to the original form of the IGT [11,17]. The back of each deck looked identical, but they differed in composition. Decks A and B were the disadvantageous decks: they yielded immediate rewards but in the long run involved major economic losses. Decks C and D were the advantageous decks: they yielded frequent small wins and smaller long term penalties, which resulted in long-term gain. The subjects were not informed of the number of trials. To adapt the IGT to our French population, the money used to play was converted from US Dollars to Euros. At the beginning of the IGT, participants had a loan of 2,000€.

**Fig 1:**
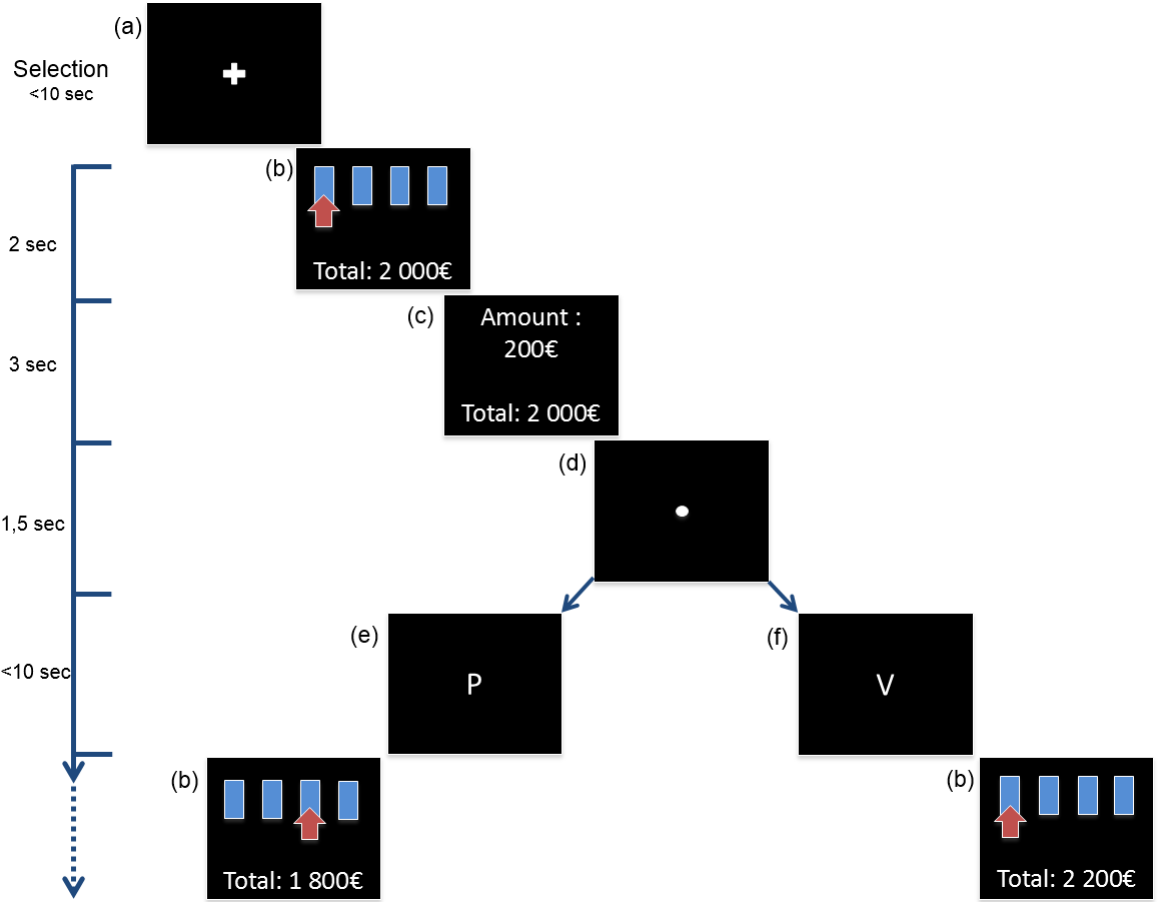
Design of the experiment. (a) For the first trial and trial following the break, subjects had to fix a cross while making their selection by pressing a key. (b) Selection was followed by a feedback of the deck chosen and the total credit amount. (c) Then the money involved in this trial was displayed. (d) A fixation point appeared to focus the eyes, followed by a fixation letter announcing the result. Half of subjects (5 men/5 women) received the information that (e) the letter P means loss (“Perdu” means loss) and (f) letter V means win (“Victoire” means victory), and the other half received the opposite information.

A few changes had to be made to adapt the IGT task to the EEG. First, to extend the electrophysiological recording of the hunch phase, the number of trials was increased from 100 to 200 trials and subjects had no hints about the presence of advantageous or disadvantageous decks. Each deck contained 200 cards. Second, the design of the trial process had been modified to minimize ocular artifacts (Fig 1). For each trial, subjects had to focus on a cross or a letter while making their selection by pressing a key. After the selection a feedback of the deck chosen and the total credit amount were displayed, followed by the amount of money involved in this trial. Then a fixation point appeared in order to focus the eyes, followed by a fixed letter announcing the result. Half of subjects (5 men/5 women) received the information that the letter P means win (“Positif” means positive) and letter V means loss (“Vaincu” means defeated), and the other half received the opposite information (“Victoire” means victory/ “Perdu” means loss). Subjects received the instruction to focus on the letter and not to blink as long as they had not made their next selection. Our choice to show a letter and not the amount of money and outcome simultaneously was to avoid ocular movements induced by reading the amount. Before beginning the task, subjects were trained with a 5-trials short version of the game.

After the task, subjects were asked which decks they thought were advantageous and disadvantageous, in order to determine if they had developed an explicit knowledge of the decks.

### Behavioral data analysis

A behavioral analysis was performed on the subjects’ performances, their reaction time and responses to questionnaires.

For the performance, the 200 trials were divided into 10 blocks of 20 trials. In each block, the net score was calculated by subtracting the number of disadvantageous decks from the number of advantageous decks selected. Hunch and conceptual phases were separated from the calculation of the net score. The last blocks where the net score remained stable were considered a part of the conceptual phase. In order to specifically examine the neural mechanisms underpinning the elaboration of a successful long-term strategy in the present task, the net scores from the conceptual phase were used to categorize participants. Indeed, it is only in that phase that some subjects developed a conscious strategy to succeed in the IGT. Subjects were classified post hoc into three group differing in net score: favorable if the net score was higher than 10, unfavorable if the net score was less than -10 and undecided if the net score range was between 10 and – 10. The use of these criteria to categorize favorable and unfavorable groups was selected from the previously reported literature on the IGT [8,11,15].

### EEG recording and data analysis

During the task, all EEG channels were recorded using the OSG digital equipment (BrainRT, OSG bvba, Rumst, Belgium) with two Schwarzer AHNS epas 44 channels amplifiers (Natus, Munich, Germany). EEG signals were acquired from 64 electrodes at the positions of the 10/10 systems using a 64-channel electrode cap (Easycap, easycapGmbh, Ammersee, Germany). EEG data were continuously recorded with a band pass of 0.05–100Hz, and a sampling rate of 1000 Hz. Signal processing was performed using Cartool Software (http:/brainmapping. unige.ch/Cartool.php).

In addition to a criteria artifact rejection of ± 100 μV, data were visually inspected in order to reject epochs with blinks, eye movements or other sources of transient noise. There were 20.7% of rejected trials for the analysis of the DPN and 23.6% for the P200/P300. Data on electrode artifacts from each participant were interpolated using a 3-dimensionalspline algorithm (average: 0.78% interpolated electrode; [48]).

Analyses were conducted during three temporal intervals:

The first temporal interval started before deck selection, while subjects were fixating on a cross or a letter, to measure the anticipatory DPN component. A time interval from 600 ms before to 200 ms after the selection was chosen on the basis of past experiments [3,28]. In agreement with previous studies the first 10 trials corresponding to the pre-punishment phase were excluded from the analysis of the anticipation component. The condition studied was defined by the choices made by the subject, i.e. if they choose advantageous (C or D) or disadvantageous (A or B) decks. For each subject both advantageous decks and disadvantageous decks were averaged.

For statistical purpose electrodes were clustered into six regions of interests. Each region comprised of seven electrodes: anterior left (F1, F3, F5, F7, AF3, AF7, FP1), anterior right (F2, F4, F6, F8, AF4, AF8, FP2), central left (C1, C3, C5, FC1, FC3, CP1, CP3), central right (C2, C4, C6, FC2, FC4, CP2, CP4), posterior left (P1, P3, P5, P7, PO3, PO7, O1), posterior right (P2, P4, P6, P8, PO4, PO8, O2).

The two other temporal intervals occurred after the result (win / loss). The first component was the P200 and was analyzed in one time window of 200–230 ms. The second component was the P300 and was analyzed in one time window of 450–500 ms. Six central electrodes (Fpz, Fz, Cz, CPz, Pz, Oz) were chosen on the basis of articles previously published in the literature regarding feedback processing [3,23–25,27,28,49].

### Source imaging

To estimate the brain regions accounting for the different electrocortical map configurations, source localization was applied using a distributed linear inverse solution based on a Local Auto-Regressive Average (LAURA) model comprising a solution space of 3005 nodes. Current distribution was calculated within the grey matter of the average brain provided by the Montreal Neurological Institute (MNI). Similar to statistical parametric mapping (SPM) used in fMRI studies, we computed the contrasts of local electrical current densities between the two anticipation conditions with time-point wise paired Student's-t-test in the periods in which the map configurations significantly differed, i.e. the period of time ranging from 600 ms before until selection. Source imaging was performed during the DPN (-600 at 0 ms), the P200 (200–230 ms) and P300 (450–500 ms) time windows. Results obtained by source imaging for groups or conditions were compared and thereby identified different regional brain activities.

## Results

### Psychometric and socio-demographic data

As evidenced by the self-rating scales, volunteers had no addictive disorders, anxiety or depression. Four healthy participants were reported to occasionally gamble. South Oaks Gambling Screen confirmed a good game control with a score of 1 for only one participant, the others having a score of 0. All subjects had scores compatible with the absence of impulsivity on the BIS-10 both generally and at sub-score level. In our group of subjects, all scores for each dimension of the Big Five Inventory were similar those conventionally found in the general population [47]. Groups did not differ either by age (t = -1.31; p = 0.21), or by educational level (t = 0.65; p = 0.62).

### Behavioral results

**Fig 2:**
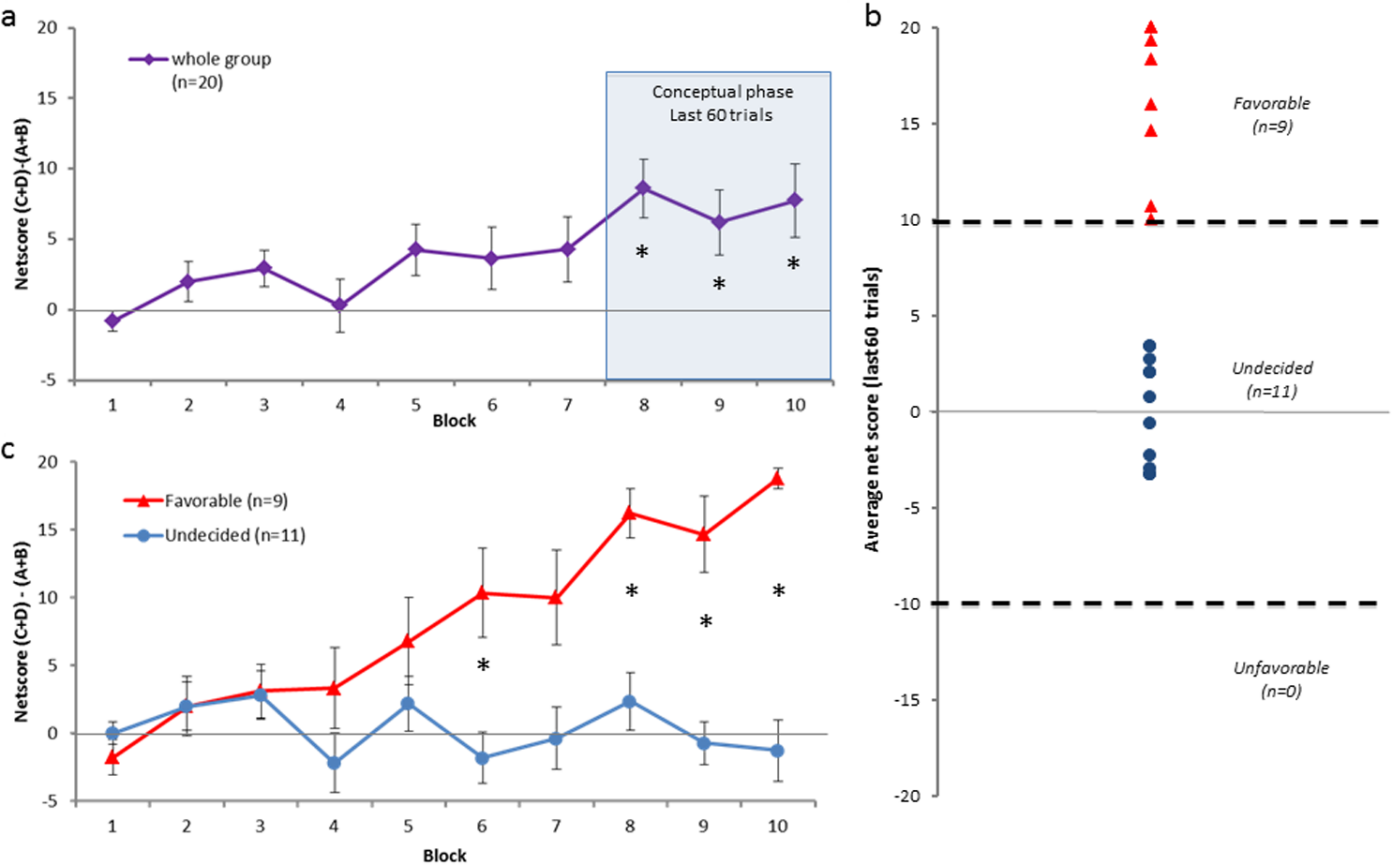
Behavioral performance in the Iowa Gambling Task. (a) Evolution of the net score in each block for the whole group. A significant difference occurred between the first block and the last 3 blocks corresponding to the conceptual phase. (b) Average net score for each subject during the conceptual phase. Two distinct populations could clearly be distinguished in this phase: Favorable and Undecided groups. (c) Evolution of the net score in each block for the Favorable and Undecided groups.

The group analysis of 20 subjects showed an increase of performances over the task, subjects playing more and more advantageous cards during the IGT. Repeated ANOVA revealed that the block effect was significant (*F*_*(9*.*171)*_ *= 3*.*7322; p < 0*.*001*). *Post-hoc* analysis revealed that the net score on block 8, 9 and 10 were significantly superior to the other blocks (from block 8, *p < 0*.*05*) (Fig 2A). Two groups were clearly distinguished: the Favorable group composed of 9 subjects who all had a score above or equal to 10 (6 male; 3 female) and the Undecided group composed of 11 subjects who all had a score between 10 and -10 (4 male; 7 female) (Fig 2B). No subject had an Unfavorable strategy, i.e. a net score below -10. Data were submitted to repeated measure analysis of variance (ANOVA), repeated measure factors being Blocks (1 to 10) and Groups (Favorable/Undecided). The Groups x Blocks effect was significant (*F*_*(9*.*162)*_ *= 7*.*4373; p < 0*.*001*). *Post-hoc* analysis revealed that Groups on block 6, 8, 9 and 10 were significantly different (from block 8, *p < 0*.*01*) (Fig 2C).

For each deck the average reaction time was calculated and compared between groups. The averaged reaction time for each deck was submitted to repeated measure analysis of variance (ANOVA), repeated measure factors were Decks (disadvantageous A and B/advantageous C and D) and Groups (Favorable/Undecided). No main difference in reaction time was found between groups but the Groups x Decks effect were significant (*F*_*(3*.*54)*_ *= 5*.*5154; p<0*.*005*). Post-hoc analysis (Tukey corrected) showed that reaction time for the selection was significantly shorter for the advantageous deck in the Favorable group (Tukey test, *p<0*.*001*), whereas no effect was noted in the Undecided group.

The answers in the questionnaire completed at the end of the task were analyzed for each sub-group, taking into account if at least one advantageous or disadvantageous deck was identified as such. The differences in the answers between sub-groups were significantly different, with the identification of at least one advantageous deck for 100% of the Favorable group as opposed to 45.4% of the Undecided group (chi^2^ = 7.01; *p* < 0.01), and the identification of at least one disadvantageous deck for 100% of the Favorable group as opposed to 63.6% of the Undecided group (chi^2^ = 4.09; *p* < 0.05). Furthermore, 80% of the subjects said they enjoyed the game, without difference between groups. Student's-t-tests were used to compare the self-rating scales in the psychometric data which did not show any significant difference between each group.

### EEG results

#### Decision preceding negativity (DPN)

Based on behavioral and literature data only trials from the pre-hunch and hunch phases were analyzed for the DPN, i.e. trials 11 to 140. Data were submitted to repeated measures analysis of variance (ANOVA), repeated measures factors being Condition (advantageous/disadvantageous), Gradient (anterior/central/posterior), Laterality (right/left) and Groups (Favorable/Undecided).

**Fig 3:**
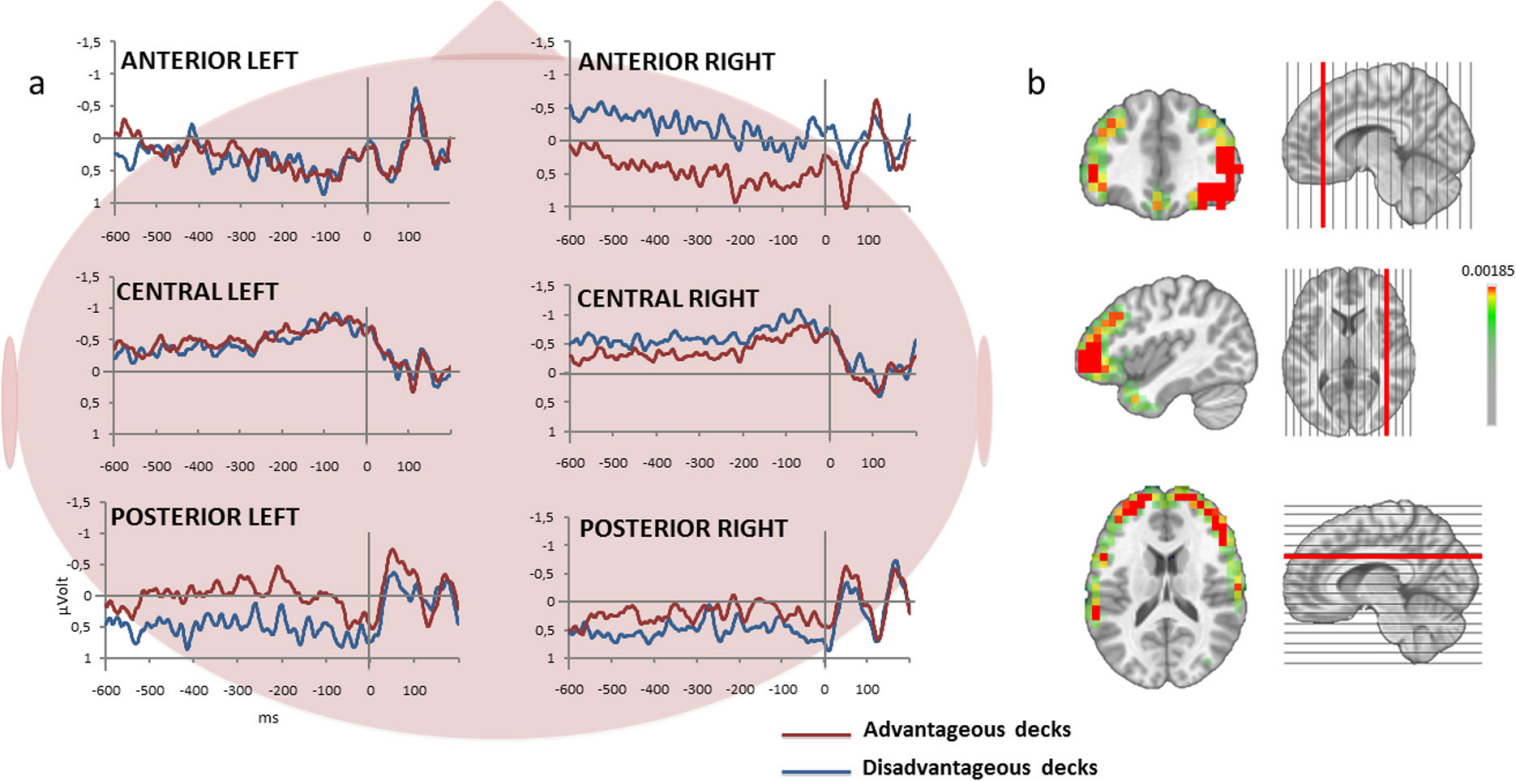
Decision preceding negativity. (a) Grand average of electrophysiological activity of the anticipation of the disadvantageous or advantageous decks on the six clusters. (b) Differences in the anticipation generation between disadvantageous and advantageous decks. A greater activity at the right frontal gyrus level was observed for the disadvantageous decks. Please note that the left side of the brain is shown on the left side of the axial MRI slides.

Analysis of the DPN revealed that disadvantageous choices differed from advantageous choices in terms of Laterality (*F*_*(1*.*19)*_ *= 15*.*044; p<0*.*001*), Gradient (*F*_*(2*.*38)*_ *= 6*.*728; p<0*.*01*) and Laterality x Gradient (*F*_*(2*.*38)*_ *= 4*.*6629; p<0*.*05*). *Post-*hoc analysis revealed that during the disadvantageous deck anticipation, a more negative potential was present in the right anterior region of interest (*p<0*.*001*), and a more positive potential in the left posterior region of interest (*p<0*.*001*) (Fig 3A).

The source localization confirmed the activation of frontal, parietal and occipital regions for both advantageous and disadvantageous conditions during anticipation (Fig 3B). To accurately determine which region was responsible for the frontal surface differences between both conditions, a Student's-t-test was performed in several regions of interests which were right and left inferior frontal gyri, right and left superior frontal gyri. A significant difference was found in the right superior frontal gyrus (*t*_*(19)*_ *= 2*.*14; p<0*.*05*). Similar tests carried out on parietal and occipital regions did not show any significant difference between both conditions.

#### Information processing

Since information processing is not influenced by the different phases of IGT, the analysis of its components was carried out on all trials, i.e. trials 0 to 200. Data were submitted to repeat measure analysis of variance (ANOVA), and repeated measure factors were Groups (i.e. Favorable/Undecided), Outcome (win/loss) and Electrodes (Fpz, Fz, Cz, CPz, Pz, Oz). Unless specified otherwise *post-hoc* tests of simple effects were Tukey corrected.

**Fig 4:**
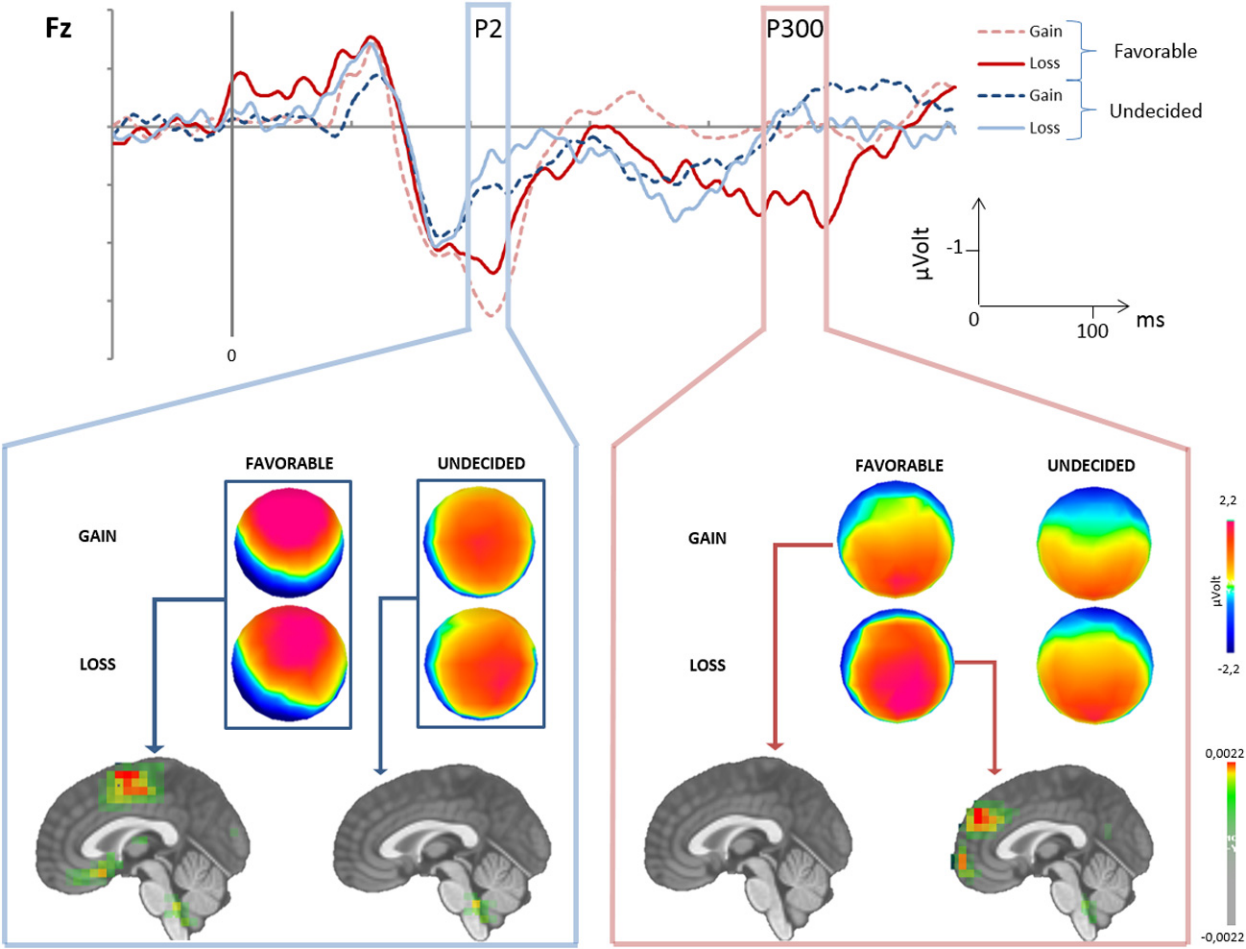
Feedback processing. Top: Feedback processing on the electrode Fz. Middle: surface topography for gain and loss in both groups of subject. Down: source imaging.

The analysis of the P200 showed that its amplitude was not modified by the Outcome (gain/loss) (*F*_*(1*,*18)*_ *= 0*.*4115; N*.*S*), by Outcome x Groups (Favorable/Undecided) (*F*_*(1*.*18)*_ *= 0*.*0846; N*.*S*), and by Outcome x Groups x Electrodes (*F*_*(5*.*90)*_ *= 0*.*5163; N*.*S*). However, significant differences were observed between Groups x Electrodes (*F*_*(5*.*90)*_ *= 6*.*8502; p < 0*.*001*). *Post-hoc* analysis revealed that the P200 was more positive for the Favorable group than for the Undecided group regarding the frontal electrodes (*LSD test; Fpz*: *p = 0*.*01; Fz*: *p = 0*.*004*) (Fig 4). These differences were confirmed by source imaging which showed an activity in the cingulate gyrus noticeable for the Favorable group alone.

The analysis of the P300 showed a significant difference between Outcome (*F*_*(1*.*18)*_ *= 14*.*098; p < 0*.*001)*, and Outcome x Groups (Favorable/Undecided) (*F*_*(1*.*18)*_ *= 5*.*1436; p = 0*.*03*). *Post-hoc* analysis revealed that the information processing was similar after a gain in the Favorable and Undecided groups as well as after a loss in the Undecided group. However, a strong positivity was observed after a loss in the Favorable group (*p < 0*.*05* compared all other conditions) (Fig 4). Source imaging applied to the Favorable group showed that the processing of a loss induced a stronger activation of the frontal medial gyrus compared to a gain.

## Discussion

Our results confirm that in a same healthy population some subjects are able to develop a successful strategy whereas others remain undecided and are unable to move toward a specific type of choice. By means of event-related potentials (ERPs), we provided evidence that these differences in performances are due to a difference in feedback processing.

To reproduce the uncertain conditions inherent to real life under laboratory conditions subjects had to perform a modified version of the IGT adapted to ERPs analysis. The analysis of the entire group of subjects showed an increase of advantageous choices during the task, which is in agreement with previous studies [11]. However, when focusing on the strategy employed on the last blocks for each participant we were able to specify whether or not these subjects reached the conceptual phase or not [17]. Thus, two distinct groups were observed, one having developed a favorable strategy and the other group who remained undecided. No subject developed a disadvantageous strategy similar to patients with VMPFC lesions. Throughout the task a learning process was only observed in the Favorable group, whereas the Undecided group showed no preference for any decks. The analysis of reaction time provides further evidence of a dichotomy between a Favorable and an Undecided group. In fact, the group who managed to develop a favorable strategy made implicit differences between decks, with a longer reaction time for disadvantageous choices. This type of difference in reaction time was not noticeable in the Undecided group. A final argument in favor of two separate strategies is provided by the questionnaire given at the end of the task. The Favorable group was able to acquire an explicit knowledge of the advantageous and disadvantageous decks whereas subjects from the Undecided group failed to identify what differentiated the entire decks. Undecided subjects appeared to be less motivated, showing a disinterest for money and gambling. These differences in strategy were previously observed in a small proportion of healthy subjects [8,29–31], and in our study it reached 55%. This increase was expected since subjects were not informed prior to the experiment that some decks were disadvantageous and others advantageous, which differs from the instructions given in the original IGT. Moreover, the absence of a hint in the instructions is associated with an increased uncertainty and a delayed onset of the conceptual phase in the IGT [50]. Furthermore, the modification of the IGT to the recording of the ERPs may also be a partial explanation for this increase. It is important to note that some studies, using a modified version of the IGT adapted to cerebral recording, did not report the existence of subjects unable to develop an adequate strategy during the task [3,28]. However, in these studies, the group analyses performed on the behavioral level did not show an increase of performance as significant as in the original version, which suggests that some subjects may have remained strategically undecided.

To understand the cerebral mechanisms underlying the development of the favorable and undecided strategies, an electrophysiological analysis of the different stages involved in decision-making was performed in both groups. Here, we specifically focused on two periods of interest, the anticipation and the feedback processing.

In the present study the anticipation of an economic decision was reflected on the cortical level by a slow cortical potential. A larger negativity was in fact found on the disadvantageous condition compared to the advantageous condition on the anterior right electrodes. This finding is in agreement with previous reported studies that associated a negative potential called DPN with the anticipation of risky choices [28]. The larger the amplitude of the DPN, the more an avoidance behavior will be generated by subjects [3]. Indeed, DPN corresponds to changes of surface electrical activity that are generated to regulate the mobilization threshold for local excitation (slow negative potential) or inhibition (slow positive potential) [51]. The specific design of the IGT used in the present study, which allowed us to perform source imaging, led to the identification of the right superior frontal gyrus in the DPN generation, thus confirming the hypothesis emitted by Bianchin et al [28]. This structure has been associated with the appreciation of the consequences of choices [16] and with doubt processes that are at the basis of intuitive judgments [52]. Furthermore, the laterality of the activation, being restrained to the right frontal area is in agreement with the relationship between emotive functions and asymmetric cortical activity [52–54]. The right frontal part of the brain is described as involved in the elaboration and control of negative emotions [53,54]; it is consistent with the right superior frontal gyrus activation that we observed in the anticipation of disadvantageous decks. However, as Favorable and Undecided groups have the same activation pattern, it can be deduced that this anticipatory process is not involved in the differences of strategic development in our healthy population. The anticipatory component of decision-making is therefore not responsible for the behavior of the Undecided group during the task.

In contrast with the DPN, the P200 analysis has shown a significant difference between Favorable and Undecided groups with a blunted neurophysiological response in subjects unable to develop an effective strategy. In agreement with studies in the previously reported literature, the cingulate gyrus was found to be involved in the P200 generation. The activity of this structure is known to be involved in stimulus-reinforcement learning and in performance monitoring [10,55–59]. If the P200 has been known to reflect early evaluation of the result on a binary classification: good or bad outcomes [3,24,25,60,61], in our study no difference of the P200 was reported for gain/loss treatment. The changes in the design of the IGT may be responsible for the lack of a significant difference in early gain and loss processing, because the amount of money and the outcome are not displayed simultaneously. The distinction between predictable and unpredictable outcomes is also known to affect the P200 and its amplitude is correlated with risk-taking [57,62]. We can hypothesize that the attenuation of the P200 in the Undecided group reflects a lower awareness in risk-taking and a reduced ability in predicting the outcomes.

In accordance with previous studies our analysis of the P300 for the entire group showed a significant difference in feedback processing [3,23,30,63,64]. We also found a higher positivity for a loss compared to a gain. This effect was specifically present in the Favorable group, whereas in the Undecided group no difference was observed between gain and loss processing. Furthermore, we confirmed that one of the generators of the P300 wave was located in the medial frontal gyrus during loss processing. [16,58,59,65,66]. In the literature the P300 was associated to performance monitoring and behavioral adaptation [63] and is influenced by attention and working memory updating [67,68]. Differences reported between Favorable and Undecided groups, thus suggest that subjects who were able to develop an efficient strategy were more attentive and vigilant to the outcome. Moreover, P300 is associated with motivational processes [69] and its amplitude is proportional to the motivational level [70,71]. It provides an additional argument explaining the lack of P300 in the Undecided group whose motivation may be reduced compared to the advantageous group, with repercussions for working memory and attention.

This result is important for studies investigating the P300 following positive and negative outcome in uncertain decision making. Indeed, if several studies reported differences in strategies in healthy population [8,30], the analyses of the P300 were always performed on the entire group and thus included subjects unable to develop a significant waveform [3,30]. The analysis of the P300 following an economic feedback should take into account the different strategies developed by the participants.

A possible limitation of this study lies in the difference in the number of men and women respectively in favorable and undecided groups, with an increased proportion of women in the undecided group. Whether this difference of representation could have affected our results is unclear and the literature is contradictory about the effect of gender on the performance at decision-making paradigms [72]. In the standard version of the IGT, some differences have been observed between men and women on the deck choice [73]. However, the overall performance is usually similar for both groups [74–77] and there is about the same probability to find good and poor performing men and women after 100 trials [77]. However, a recent review suggests that there are some differences in the range 60–100 trials, where women need more trials before preferring to select long-term advantageous decks [77]. As a modified version of the IGT was used in the present study, it is difficult to know whether similar differences occur and whether gender has impacted the elaboration of a strategy. Since our groups of participants were selected post hoc, it was not possible to control this parameter. Further studies are thus needed to explore whether the neural bases involved in the elaboration of a strategy are influenced by gender. The resolution of this question will help the interpretation of the results presented here. Indeed, gender has been identified as a significant predictor of the motivation level, men appearing more motivated than women [78], and differences in motivation levels between genders could also explain the neurophysiological discrepancies observed in the present study.

In summary, feedback processing is a complex and influenced by several factors such as motivation and emotion [64]. The quality of this processing is related to explicit cognitive components. In contrast the lack of difference between Favorable and Undecided groups on the process of anticipation shows that there is no influence of motivation on the anticipatory component. These results are consistent with the somatic markers hypothesis and confirm the persistence of a normal anticipation activity in healthy subjects with poor strategy, a phenomenon already observed by electrodermal studies [8,17]. Anticipation is an implicit unconscious and automatic process, influenced by the somatic markers.

To conclude, our behavioral results confirmed the presence of different strategies in the IGT that cannot be explained by socio-demographic differences or personality. The cerebral mechanisms involved in the evaluation of the choice remains similar whatever the efficacy of the strategy adopted. An attenuated feedback processing was observed in the Undecided group that could explain why this group did not reach the conceptual phase. The different stages of information processing suggest that Undecided subjects are hyposensitive to valence during gambling, which is in opposition to problem gamblers who are hypersensitive to rewards [26]. The capacity of whether or not to elaborate a strategy in the healthy population may be explained by the subjects’ motivation to gamble and attention. Uncertainty in the IGT therefore does not constitute a vulnerability of factors to any neuropsychiatric disorders. The motivational processes have an important role in decision-making by helping to make a choice among the various alternative options [79]. These factors may be a prerequisite to the development of an effective strategy in uncertain decision-making.
